# Knowledge, Attitudes, and Practices among Dental Practitioners Regarding Antibiotic Prescriptions for Pregnant and Breastfeeding Women in the Dominican Republic

**DOI:** 10.3390/antibiotics10060668

**Published:** 2021-06-03

**Authors:** Javier Aragoneses, Ana Suárez, Cinthia Rodríguez, Juan Algar, Juan Manuel Aragoneses

**Affiliations:** 1 Department of Medicine and Medical Specialties, Faculty of Health Sciences, University of Alcalá, 28801 Madrid, Spain; javias511@gmail.com; 2 Department of Preclinical Dentistry, School of Biomedical Sciences, Universidad Europea de Madrid, Villaviciosa de Odón, 28670 Madrid, Spain; 3 Department of Dentistry, Universidad Federico Henriquez y Carvajal, 10106 Santo Domingo, Dominican Republic; cinthiagarabitos@gmail.com; 4 Department of Clinical Dentistry, School of Biomedical Sciences, Universidad Europea de Madrid, 28005 Madrid, Spain; juan.algar2@universidadeuropea.es; 5 Dean of The Faculty of Dentistry, Universidad Alfonso X El Sabio, 28691 Madrid, Spain; jmaragoneses@gmail.com

**Keywords:** antibiotics, KAP survey, Dominican Republic, pregnant, breastfeeding

## Abstract

In this study, we aimed to explore the trends among dentists in the Dominican Republic of providing antibiotic prescriptions to pregnant/breastfeeding dental patients. A survey was conducted among 98 dentists, using a self-administered questionnaire, about their knowledge and attitudes regarding antibiotic usage in pregnant/lactating women and the translation of these into practice. The majority of the survey population were female dentists (63.3%) aged 45–54 years. A chi-square test showed statistically significant differences in the knowledge sources between older and younger dentists, with a minority having chosen scientific literature as a source (*p*-value of 0.04). There were statistically significant associations between gender and certain attitudes and practice-based questions, with *p*-values of 0.04 and 0.01, respectively. The Spearman’s correlation test showed a statistically significant correlation between knowledge and attitude (*p*-value 0.001), whereas no correlation was found with practice (*p*-value 0.23). A multiple response analysis showed that the majority of the respondents chose the second and third trimester for antibiotic prescriptions for acute conditions such as cellulitis, periodontal abscess, and pericoronitis. Most dentists had sufficient knowledge about antibiotic usage in pregnant/lactating women, but it did not translate into practice, and a certain proportion of the participants followed incongruent drug prescription. These findings can be used to focus on judicious antibiotic usage by dentists in the Dominican Republic.

## 1. Introduction

Resistance to antimicrobial drugs poses a serious threat to human life and may result in detrimental economic and public health burdens [1]. Antibiotic resistance can be due to the following: (i) bacterial mutation and subsequent selective adaptation of bacteria to sublethal exposure to drugs and (ii) spread of mobile genetic elements (e.g., plasmids and transposons) by the recombination process. As a result, bacteria no longer respond to antibiotics that can kill or potentially inhibit their growth. It is imperative to control the spread of antibiotic resistance, since it has been observed that, by the time the resistant trait is identified, it has already spread widely in nature [2]. The resistance is due to antibiotic resistance genes (ARGs), which can be found in bacterial communities existing in various environments (food, soil, and waterways) as well as the human body. These communities are also known as microbiomes, and they consist of an ecosystem made of commensals, symbionts, and pathogenic microorganisms. ARGs, also known as resistomes, can be found within this ecosystem [3].

It has been reported that 40–50% of antibiotic prescriptions worldwide are unwarranted, and dentistry accounts for about 3–11% of these prescriptions [4]. With clinicians significantly contributing to this trend, there is an impetus for understanding the ramifications of clinical decisions beyond the dental chair [5]. It has been observed that about 66% of the antibiotics prescribed in a dental setting may not have an indication. For disease control, prevention is a key factor, and dentists can fulfil their responsibility for controlling antibiotic resistance by adhering to the following guidelines: (i) achieve an accurate diagnosis before antibiotic prescription; (ii) use appropriate antibiotics for the presenting clinical diagnosis based on the microbiology; (iii) use a narrow spectrum of antibiotics, where possible [6,7].

Common infections during pregnancy are treated through antibiotics to prevent maternal or neonatal infections during delivery or postpartum following cesarean surgery [8,9]. Studies conducted in Canada and the Netherlands have reported that about 20–25% of women receive antibiotics at some point during their pregnancy [10,11]. In addition, it has been shown that one in five women in Europe has been prescribed at least one antibiotic during pregnancy, and the rate is double that in the United States [12]. However, it is prudent to note that a decision to prescribe antibiotics in pregnant women would follow a risk–benefit analysis [13].

Good oral health and control of disease can increase the quality of life and protect women’s general health. In turn, pathogenic organisms have the potential to be transmitted from mother to child. Some antibiotic groups are contraindicated during pregnancy and breastfeeding due to major side effects [14]. These side effects include alterations in the bowel and gut flora and direct effects that may or may not be dose dependent [15]. The most commonly used antibiotics in dentistry are penicillins (such as amoxicillin and ampicillin) and cephalosporins (such as cephalexin and cefaclor), which are generally considered to be safe during pregnancy. Traditionally, tetracyclines have been contraindicated in pregnant and lactating women because of the possibility of permanent tooth staining in the developing fetus and because of the risk of hepatotoxicity in pregnant women. Second-generation tetracyclines, such as doxycycline, have fewer side effects, though it is unclear whether they can cause permanent tooth staining when prescribed for pregnant women [16]. The other drugs to be used with caution following a risk–benefit analysis include fluoroquinolones, macrolides (excluding azithromycin), polymyxins, and oxazolidinones [14].

A Centers for Disease Control and Prevention study, based on a survey, reported that, in general, breastfeeding occurred in the early postpartum period, six months, and twelve months following delivery at percentages of 81.9, 60.6, and 34.1, respectively [15]. Drug prescriptions for such patients are reliant on the drug concentration in the milk; in most cases, the drugs considered acceptable during pregnancy can also be used during lactation. However, some drugs have a small molecular size, and the concentration can become high in breast milk; some also have a longer half-life period [17,18]. Many antibiotics, such as amoxicillin, ampicillin, azithromycin, and cephalexin, can be safely used during breastfeeding, but caution has to be exercised regarding the use of fluoroquinolones, erythromycin, tetraycline, and clotrimoxazole, as they were found to be in higher concentrations in breast milk, with increased potential for adverse effects [19].

Antibiotic drug therapy continues to be an essential therapeutic component in dentistry. Although guidelines are available, dentists may not always be aware of the current regulations regarding antibiotic prescriptions [20]. According to the literature on dentistry, antibiotics should be prescribed to healthy patients as a prophylactic measure in cases of surgery for the removal of benign tumors, exodontia of impacted teeth, surgery for the placement of implants, bone grafting, and periapical surgery. Antibiotics are also recommended for acute infections such as ulceronecrotising gingivitis, aggressive localized periodontitis, periodontal abscess, acute apical abscess, peri-implantitis, pericoronitis, and cellulitis, among others [20,21,22,23,24].

For pregnant and breastfeeding women, the same indications should be applied as for non-pregnant and non-lactating women, taking into account that the antibiotic of choice should not cause any alterations in the fetus or the infant [21,25].

The development of resistance to antibiotic drugs is a natural phenomenon, but it can become accentuated due to improper usage or misuse of antibiotics [26]. The prevention of antibiotic misuse can have global implications for preventing the development and spread of antibiotic-resistant bacterial strains [20]. The present study aimed to understand the knowledge, attitudes, and practices among dentists in the Dominican Republic regarding providing antibiotic prescriptions to pregnant and breastfeeding dental patients.

## 2. Results

In this study, a knowledge, attitude, and practice (KAP) survey was conducted among 98 dental practitioners; the majority of the respondents were female dentists, i.e., 63.3% (*n* = 62). Among the female dentists, the majority of the participants (*n* = 25) belonged to the 45–54 age group, followed by those (*n* = 22) who belonged to the 35–44 age group. Nearly half of the respondents among the male dentists belonged to the 25–34, 55–64, and over 65 years age groups (Table 1).

There were no statistically significant differences observed among the different age groups and their responses to the attitude and practice questions on antibiotic prescriptions in pregnant and breastfeeding women. There was a statistically significant difference (*p*-value < 0.04) observed for a knowledge-based question that assessed the sources of the dentists’ knowledge about prescriptions of antibiotics to breastfeeding women; 75% of the over 65 years age group responded that they acquired their knowledge during undergraduate studies, whereas 33.3% of the 55–64 age group acquired their knowledge from the scientific literature. About 64.3% of the predominantly younger population of dentists (25–34 years) answered that they obtained their knowledge from specialized websites (Table 2).

From the chi-square test, it can be observed that there are statistically significant differences according to the gender of the study population in their responses regarding attitude and the practice of providing antibiotic prescriptions to pregnant and breastfeeding women. About 94.4% of the male dentists prescribed antibiotics to breastfeeding women as compared with 30.6% of their female counterparts (*p*-value of 0.04). There were statistically significant differences between the male and female study populations based on the practice question on the type of antibiotic prescribed to pregnant women, with a *p*-value of 0.01. Nearly half of the male dentists (47.2%) prescribed amoxicillin 875 mg + clavulanic acid, followed by 38.9% who prescribed amoxicillin 500 mg; the least prescribed antibiotic was clindamycin (300 mg) at 2.8%. Some male dentists did not recommend prescribing metronidazole (500 mg) or clindamycin (600 mg) to pregnant women (Table 3).

The antibiotic of choice for pregnant women was amoxicillin (500 mg), chosen by more than half (58.1%) of the female dentist study population. It was followed by amoxicillin (875 mg) plus clavulanic acid, which was recommended by 16.1% of female dentists. The least recommended antibiotics for pregnant women among the female dentists were azithromycin (500 mg) (3.2%), metronidazole (500 mg) (3.2%), and clindamycin (600 mg) (1.6%). Another noteworthy finding was that 12.6% of the female dentists did not have an answer on the choice of antibiotics for pregnant women as compared with 2.8% of their male counterparts (Table 3). The Spearman’s correlation test observed that there was a statistically significant correlation between knowledge and attitude scores, with a correlation coefficient (rho) of 0.34 and *p*-value of 0.001; however, a correlation coefficient value of 0.34 is considered to be a weak correlation. There was no significant correlation between knowledge and practice scores (Table 4).

The percentage distribution and multiple response analysis were performed for two knowledge-based questions that had multiple choice answers that the dentists could select. The questions consisted of the choice of trimesters considered to be safe for antibiotic prescriptions in pregnant women and the treatments for which antibiotic drugs would be prescribed to pregnant women. It was observed that 88.9% of the respondents chose the second trimester, which constituted about 38.1% of all the responses, followed by the third trimester, chosen by 80% of the respondents and constituting 34.3% of all the answers to the question. Another finding was that 55.6% of the respondents chose the first trimester, which constituted about 23.8% of the answers pertaining to this knowledge-based question. The recommended diagnosis/treatments for antibiotic prescription in pregnant women chosen by 90.7% of the respondents was cellulitis, followed by endodontics in teeth with pulp necrosis and acute apical abscess with/without systemic involvement, chosen by 83.5% of the respondents. The least preferred treatment for antibiotic prescription was scaling and root planing, chosen by 6.2% of the respondents and constituting 1.5% of the answers (Table 5).

When the proportions of dentists prescribing antibiotics for pathologies most frequently associated with systemic symptoms were compared with the trimester of pregnancy using a logistic regression model for repeated measures, no differences were observed between antibiotic prescription and trimester of pregnancy (Table 6).

## 3. Discussion

This study represents one of the few attempts in the existing literature to assess the knowledge, attitudes, and practices of dental practitioners regarding providing antibiotic prescriptions to pregnant and breastfeeding women and the first survey to be conducted in the Dominican Republic. In this survey, the majority of the respondents were female (63.3%) and most of them belonged to the 45–54 age group. The male respondents were almost equally distributed among the 25–34, 55–64, and over 65 years age groups.

A significant finding from the associations between age and KAP questions was that older dentists relied on their knowledge acquired during undergraduate studies, whereas younger dentists updated their knowledge about prescribing antibiotics using specialized websites. Only a small proportion of the dentist population obtained their knowledge from scientific literary sources. In contrast, authors such as Abukaraky et al. [27] observed that the vast majority of dentists obtained information about antibiotic prescribing both through knowledge from reading books, articles, or the Internet as well as from basic and specialized education. Chen et al. [28] in 2020 observed a difference between dentists, who followed prescribing guidelines as a source of knowledge, and oral and maxillofacial surgeons, who relied on knowledge acquired during their specialized training. However, none of these articles made an association with age. A review of the evidence-based dentistry (EBD) approach identified that the three main avenues for knowledge translation were from primary studies such as randomized controlled trials, from systematic reviews, and through third-generation knowledge based on practice guidelines and decision aids [29,30,31]. Considering the three pillars of EBD, which include best current evidence, the expertise of the clinician, and patient’s values and preferences, it is reasonable to suppose that the older/younger dentists from this survey have not updated their knowledge through scientific literature [32].

A significant majority of the male dentists in the study population (94.4%) advised prescribing antibiotics to lactating women, while only about 30.6% of the female dentists concurred with this decision. The Food and Drug Administration (FDA) lists the commonly used antibiotics in dentistry, such as amoxicillin, azithromycin, clindamycin, cephalexin, metronidazole, and other penicillin drugs in the pregnancy risk factor category B (no evidence of risk in humans); the American Academy of Pediatrics (AAP) considers these drugs, except metronidazole, to be compatible with lactation. It has been reported that metronidazole is considered to be an in vitro mutagen, and a mother should discontinue breastfeeding for about 12–24 h following a single-dose therapy of the drug [33]. Other drugs, such as erythromycin and tetracycline, are not recommended during pregnancy/lactation due to their adverse effects [34,35,36]. Although the antibiotics mentioned in this survey for breastfeeding do not conform with the contraindicated drugs, except for metronidazole, the hesitancy among the female dentists could be attributed to the numerous potential side effects associated with antibiotic usage during lactation.

A KAP survey conducted among Lebanese dentists reported that the first-choice antibiotic drug for pregnant women was amoxicillin + clavulanic acid, followed by spiramycin and amoxicillin. Other drugs included azithromycin, clindamycin, cephalosporin, etc., and similar trends were observed for the question on providing antibiotic prescriptions to breastfeeding women [37]. Penicillins, namely amoxicillin, were the primary antibiotics prescribed by dentists in the Dominican Republic for pregnant/lactating women. Although few in numbers, some female dentists chose metronidazole as the first choice for these women, which is an alarming finding as the first-line usage of this drug is not recommended during the first trimester of pregnancy as well as during lactation [38]. It is also striking to note that a significant proportion of the male and female dentists did not have an answer regarding a drug choice for pregnant as well as lactating women, and it is reasonable to suppose that these dentists never prescribed antibiotics to this group of dental patients.

In the multiple-choice analysis, although the majority of respondents chose the second or third trimester of pregnancy as safe for antibiotic prescription, there was also a significant percentage of dentists (55.6%), which constituted 23.8% of the responses, who answered the first trimester as safe for antibiotic usage. The literature states that up to 43% of pregnant women may suffer from pathologies of odontogenic origin that can be dangerous for both the pregnant woman and the fetus because they can rapidly progress to systemic infections [39,40,41], and they should be treated aggressively to avoid further complications [41]. If drainage of the infection is not possible or there is severe inflammation or systemic involvement such as fever, antimicrobial treatment should be chosen [39], always weighing the risk and benefit to the mother and fetus [39,40,42,43], regardless of the trimester of gestation [44]. However, there is a consensus that antibiotic prescription during the first trimester of pregnancy should be avoided where possible, as it is linked to iatrogenic teratogenicity [38]. Drugs such as streptomycin, tetracycline, and kanamycin are best avoided as they have been associated with this complication [45]. The results in the present study showed an increase of prescription in the case of pathologies that more frequently present systemic implications [41,46,47,48,49,50]; nonetheless, no significant differences between trimesters regarding antibiotic prescription were present. This finding regarding indiscriminate antibiotic use by the surveyed dentists in the Dominican Republic is concerning. These results, however, have to be approached taking into consideration two main limitations. The first one is that this survey did not specify the systemic symptoms related to the ailment, and the second is that no background regarding the possibility of surgical intervention and its outcome was given. In this regard, further studies should give a more specific clinical background to the participants.

In dentistry, it is generally recommended to prescribe antibiotics as an adjunct to surgical intervention, such as debridement or drainage, for preventing the spread of infection due to systemic involvement or when the dental patient is medically compromised, with a risk of significant complications [5]. Most elective dental procedures can be postponed in a pregnant dental patient; however, those presenting with pain or an advanced stage of infection should not be delayed. It is prudent to note that none of the drugs prescribed for pain/infection are risk free, but the impact of not treating an infection can have a significant bearing on a pregnant dental patient, with potential risks involved [14].

In this KAP survey, the results of the multiple-choice analysis showed that the majority of the respondents advised prescribing antibiotics to pregnant dental patients for cellulitis (90.7%), endodontics with pulpal necrosis, acute apical abscess with/without systemic involvement (83.5%), periodontal abscess (70.1%), and pericoronitis (53.6%). An apt example of inappropriate antibiotic usage is during the treatment of acute endodontic infections without the initiation of endodontic therapy for pain alleviation. There is reduced blood circulation within the root canal in pulpal pathologies, and antibiotics are not able to target the pathogens within the pulp; however, dentists continue with the prescription of antibiotics to treat localized infections without systemic involvement [46,51,52]. The highest rates have been observed in Croatia (80%), and the lowest in the United States (33%); in this KAP survey, it was observed that 83.5% of respondents suggested antibiotic usage for endodontic infections with/without systemic involvement. Another deviant practice observed in this study was the prescription of antibiotics for scaling and root planing and simple exodontia for pregnant dental patients, and this cannot be overlooked.

This KAP survey has its limitations. Extrapolation of the results is complicated because, at the time of the survey, there was no official census available on the number of practicing dentists in the Dominican Republic; therefore, generalization of the results should be taken with caution. However, the merits of this study include being the first of its kind to be conducted in the country and among the few studies around the world to assess the trends of providing antibiotic prescriptions to pregnant/lactating women. The results of this study show that there is a negligible correlation between knowledge and practice as well as a weak correlation with attitudes among the dentists in the study population. A plausible bias due to variability in relation to gender, age, and year of graduation leads us to assert that the final assessments of this study should be made with caution. Future studies should aim at corroborating the findings with dental patient records to ensure validity.

## 4. Materials and Methods

This descriptive study was conducted among dentists in Santo Domingo, Dominican Republic, who attended a dental conference organized by the Federico Henríquez y Carvajal University in 2016. The study was conducted in the form of a KAP-based questionnaire. The study was approved by the Federico Henríquez y Carvajal University’s Ethics Committee (8 February 2016). The dental practitioners (*n* = 98) were attending a training course at the university, and they voluntarily enrolled in this study following a brief presentation about the study’s aim and protocol. Informed consent was obtained prior to participation in the study.

An eight-item, close-ended, self-administered questionnaire assessing the knowledge, attitudes, and practices among dentists regarding providing antibiotic prescriptions during pregnancy and breastfeeding to dental patients without systemic disorders was utilized in this study. The questionnaire consisted of the following: four questions on knowledge, two questions on attitude, and two questions on practices. The participants were asked several yes/no questions along with items that warranted multiple responses for a single question. The questionnaire also collected information on the age and gender of the study participants. The age range was divided into five categories: (a) 25–34 years, (b) 35–44 years, (c) 45–54 years, and (d) 55–64 years; (e) those greater than 65 years were grouped as elderly individuals.

### Statistical Analysis

The responses were collected, and the data were entered into a Microsoft Excel spreadsheet and analyzed using SPSS software (IBM, SPSS Statistics, Version 20.0, Armonk, New York, USA). Descriptive statistics were used for summarization and presentation. The Shapiro–Wilks test was performed to test the normality of the data set, and it followed a non-parametric distribution. The chi-square test was used to find the associations among the variables, and a *p*-value < 0.05 was considered to be statistically significant. The Spearman’s correlation test was used to assess the correlation between knowledge, attitude, and practice aspects of the research. A multiple response analysis was performed for the knowledge-based multiple-choice questions.

## 5. Conclusions

On the basis of a KAP survey among dentists in the Dominican Republic regarding antibiotic prescriptions to pregnant/breastfeeding women, we conclude that the majority of the dentists obtain their knowledge from their undergraduate education or specialized websites rather than the scientific literature. Although the majority of respondents conformed to the guidelines on antibiotic usage, a significant number of dentists’ responses corresponded with inappropriate usage. Penicillin was the antibiotic commonly prescribed to pregnant/lactating women among dental practitioners. There was a significant gap between knowledge and practice, and future studies should target determinants of poor knowledge and deviant practices, which do not result in clinical benefit to a dental patient.

## Figures and Tables

**Table 1:** Descriptive statistics on the distribution of study participants based on age and gender.

Age Groups	Gender		Total, N (%)
	Male, N (%)	Female, N (%)	
25–34 years	7 (50.0)	7 (50.0)	14 (100.0)
35–44 years	15 (40.5)	22 (59.5)	37 (100.0)
45–54 years	6 (19.4)	25 (80.6)	31 (100.0)
55–64 years	6 (50.0)	6 (50.0)	12 (100.0)
over 65 years	2 (50.0)	2 (50.0)	4 (100.0)
Total	36 (36.7)	62 (63.3)	98 (100.0)

**Table 2:** Chi-squared test for associations among different age groups and knowledge, attitude, and practice questions.

Questions		Age Group in Years, N (%)					Chi-Square Value	*p*-Value
		25–34	35–44	45–54	55–64	Over 65		
What knowledge do you rely on for prescribing antibiotics to pregnant women?	Knowledge acquired during undergraduate studies	8 (57.1)	26 (70.3)	24 (77.4)	7 (58.3)	4 (100)	7.29	0.50
	Knowledge of scientific literature	4 (28.6)	10 (27.0)	4 (12.9)	4 (33.3)	0 (0)		
	Specialized websites	2 (14.3)	1(2.7)	3 (9.7)	1 (8.3)	0 (0)		
On what knowledge do you base the prescription of antibiotics to breastfeeding women?	Knowledge acquired during undergraduate studies	2 (14.3)	17 (45.9)	17 (54.8)	4 (33.3)	3 (75.0)	15.63	0.04 *
	Knowledge of scientific literature	3 (21.4)	2 (5.4)	6 (19.4)	4 (33.3)	0 (0)		
	Specialized websites	9 (64.3)	18 (48.6)	8 (25.8)	4 (33.3)	1 (25.0)		
Do you always take into account in the medical history whether a woman is breastfeeding?	Yes	11 (78.6)	30 (81.1)	20 (64.5)	8 (66.7)	3 (75.0)	2.85	0.58
	No	3 (21.4)	7 (18.9)	11 (35.5)	4 (33.3)	1 (25.0)		
Do you prescribe antibiotics to breastfeeding women?	Yes	12 (85.7)	27 (73.0)	26 (83.9)	10 (83.3)	2 (50.0)	3.73	0.44
	No	2 (14.3)	10 (27.0)	5 (16.1)	2 (16.7)	2 (50.0)		
Which of the following antibiotics do you usually prescribe, if necessary, to pregnant women?	Amoxicillin 500 mg	6 (42.9)	13 (35.1)	22 (71.0)	7 (58.3)	2 (50.0)	22.82	0.53
	Azithromycin 500 mg	2 (14.3)	2 (5.4)	1 (3.2)	0 (0)	0 (0)		
	Amoxicillin 875 mg + clavulanic acid	6 (42.9)	13 (35.1)	3 (9.7)	4 (33.3)	1 (25.0)		
	Clindamycin 300 mg	0 (0)	3 (8.1)	1 (3.2)	0 (0)	0 (0)		
	Clindamycin 600 mg	0 (0)	0 (0)	1 (3.2)	0 (0)	0 (0)		
	Metronidazole 500 mg	0 (0)	1 (2.7)	1 (3.2)	0 (0)	0 (0)		
	No answer	0 (0)	5 (13.5)	2 (6.5)	1 (8.3)	1 (25.0)		
Which of the following antibiotics do you usually prescribe, if necessary, to breastfeeding women?	Amoxicillin 500 mg	7 (50.0)	18 (48.6)	21 (67.7)	9 (75.0)	1 (25.0)	33.10	0.10
	Azithromycin 500 mg	1 (7.1)	0 (0)	1 (3.2)	0 (0)	0 (0)		
	Amoxicillin 875 mg + clavulanic acid	0 (0)	10 (27.0)	1 (3.2)	2 (16.7)	3 (75.0)		
	Clindamycin 300 mg	0 (0)	1 (2.7)	0 (0)	0 (0)	0 (0)		
	Clindamycin 600 mg	0 (0)	0 (0)	1 (3.2)	0 (0)	0 (0)		
	Metronidazole 500 mg	0 (0)	0 (0)	1 (3.2)	0 (0)	0 (0)		
	No answer	6 (42.9)	8 (21.6)	6 (19.4)	1 (8.3)	0 (0)		

* Statistical significant differences.

**Table 3:** Chi-square test for associations between gender and knowledge, attitude, and practice questions.

Questions		Gender, N (%)		Chi-Square Value	*p*-Value
		Male	Female		
What knowledge do you rely on for prescribing antibiotics to pregnant women?	Knowledge acquired during undergraduate studies	25 (69.4)	44 (71.0)	0.12	0.94
	Knowledge of scientific literature	8 (22.2)	14 (22.6)		
	Specialized websites	3 (8.3)	4 (6.5)		
On what knowledge do you base the prescription of antibiotics to breastfeeding women?	Knowledge acquired during undergraduate studies	18 (50.0)	25 (40.3)	0.86	0.64
	Knowledge of scientific literature	5 (13.9)	10 (16.1)		
	Specialized websites	13 (36.1)	27 (43.5)		
Do you always take into account in the medical history whether a woman is breastfeeding?	Yes	23 (63.9)	49 (79.0)	2.68	0.10
	No	13 (36.1)	13 (21.0)		
Do you prescribe antibiotics to breastfeeding women?	Yes	34 (94.4)	43 (69.4)	8.51	0.04
	No	2 (5.6)	19 (30.6)		
Which of the following antibiotics do you usually prescribe, if necessary, to pregnant women?	Amoxicillin 500 mg	14 (38.9)	36 (58.1)	15.32	0.01 *
	Azithromycin 500 mg	3 (8.3)	2 (3.2)		
	Amoxicillin 875 mg + clavulanic acid	17 (47.2)	10 (16.1)		
	Clindamycin 300 mg	1 (2.8)	3 (4.8)		
	Clindamycin 600 mg	0 (0.0)	1 (1.6)		
	Metronidazole 500 mg	0 (0.0)	2 (3.2)		
	No answer	1 (2.8)	8 (12.9)		
Which of the following antibiotics do you usually prescribe, if necessary, to breastfeeding women?	Amoxicillin 500 mg	18 (50.0)	38 (61.3)	8.11	0.23
	Azithromycin 500 mg	2 (5.6)	0 (0)		
	Amoxicillin 875 mg + clavulanic acid	5 (13.9)	11 (17.7)		
	Clindamycin 300 mg	1 (2.8)	0 (0)		
	Clindamycin 600 mg	0 (0)	1 (1.6)		
	Metronidazole 500 mg	0 (0)	1 (1.6)		
	No answer	10 (27.8)	11 (17.7)		

* Statistical significant differences.

**Table 4:** Spearman’s correlation test for correlations among knowledge, attitude, and practice scores.

Correlations among Knowledge, Attitude, and Practice			Knowledge Score	Attitude Score	Practice Score
Spearman’s rho	Knowledge score	Correlation Coefficient (r)	1.000	0.34	0.12
		*p*-Value (2-tailed test)	0.0	0.001 *	0.23
		N	98	98	98

* Statistical significant differences.

**Table 5:** Percentage distribution of responses and multiple response analysis for the knowledge questions.

Question		Responses		Percent of Cases (Multiple Response)
		N	Percent	
If you have to give antibiotics to a pregnant woman, in which trimester do you prescribe them?	First trimester	50	23.8%	55.6%
	Second trimester	80	38.1%	88.9%
	Third trimester	72	34.3%	80.0%
	No answer	8	3.8%	8.9%
Total		210	100.0%	233.3%
For which treatments do you prescribe antibiotics to pregnant patients?	Endodontics in teeth with symptomatic irreversible pulpitis and with/without symptomatic apical periodontitis	31	7.8%	32.0%
	Endodontics in teeth with pulp necrosis and with/without symptomatic apical periodontitis	37	9.3%	38.1%
	Endodontics in teeth with necrosis and periapical granuloma	6	1.5%	6.2%
	Endodontics in teeth with pulp necrosis and acute apical abscess with/without systemic involvement	81	20.5%	83.5%
	Simple exodontia	19	4.8%	19.6%
	Pericoronitis	52	13.1%	53.6%
	Cellulitis	88	22.2%	90.7%
	Scaling and root planning	6	1.5%	6.2%
	Periodontal abscess	68	17.2%	70.1%
	No answer	8	2.0%	8.2%
Total		396	100.0%	408.2%

**Table 6:** Percentage of dentists who prescribed antibiotics according to trimester of pregnancy in pathologies that most frequently cause systemic symptomatology.

	1st Trimester		2nd Trimester		3rd Trimester		*p*-Value
Endodontics in teeth with pulp necrosis and acute apical abscess with/without systemic involvement	N	%	N	%	N	%	0.955
No	6	12	7	8.6	7	9.6	
Yes	44	88	74	91.4	66	90.4	
Cellulitis	N	%	N	%	N	%	0.999
No	1	2	1	1.2	-	-	
Yes	49	98	80	98.8	73	100	
Periodontal abscess	N	%	N	%	N	%	0.898
No	11	22	20	24.7	15	20.5	
Yes	39	78	61	75.3	58	79.5	
Pericoronitis	N	%	N	%	N	%	0.99
No	21	42	32	39.5	30	41.1	
Yes	29	58	49	60.5	43	58.9	
